# Acupotomy for patients with tarsal tunnel syndrome: A protocol for systematic review and meta analysis

**DOI:** 10.1097/MD.0000000000022369

**Published:** 2020-09-25

**Authors:** Xiaojie Sun, Qiaoyin Zhou, Chong Shi, Yangjing Lan, Yan Jia, Zuyun Qiu, Yifeng Shen, Shiliang Li

**Affiliations:** a Beijing University of Chinese Medicine; b Department of Acupuncture-Moxibustion, China-Japan Friendship hospital, Beijing; c Fujian University of Traditional Chinese Medicine; d Key Laboratory of Orthopedics & Traumatology of Traditional Chinese Medicine and Rehabilitation (Fujian university of TCM), Ministry of Education, Fuzhou, Fujian; e Hospital of Chengdu University of Traditional Chinese Medicine, Chengdu, Sichuan Province, P. R. China.

**Keywords:** acupotomy, protocol, systematic review, tarsal tunnel syndrome

## Abstract

**Background:**

Tarsal tunnel syndrome (TTS) is a painful condition of the ankle that affects patients’ quality of life and ability to work. Multiple clinical studies of nerve decompression by acupotomy have been published in China, and the results are encouraging. However, the efficacy and security of this treatment have not been evaluated scientifically and systematically. The purpose of this systematic review protocol is to evaluate the efficacy and security of acupotomy treatment in patients with TTS, which will be helpful to clinical acupotomy doctors.

**Methods:**

Relevant randomized controlled trials will be identified by searching 9 databases (PubMed, Embase, Cochrane Library, Chinese literature databases, the Chinese Biomedical Literature Database, China National Knowledge Infrastructure, SinoMed, Technology Journal and the Wanfang Database. Randomized controlled trials examining the use of acupotomy for TTS patients will be identified independently by 2 reviewers by searching the databases from inception to March 2020. Clinical effects will be evaluated as the primary outcome. Visual analog scale scores will be assessed as a secondary outcome. Review Manager 5.3 will be used to perform a fixed effects meta-analysis, and the evidence level will be evaluated by using the Grading of Recommendations Assessment, Development, and Evaluation framework. Continuous outcomes will be presented as mean differences or standard mean differences, while dichotomous data will be expressed as relative risks.

**Results:**

This study will evaluate the effectiveness and safety of acupotomy in the treatment of TTS in randomized controlled trials with high-quality visual analog scale and Roles and Maudsley score.

**Conclusion:**

This systematic review will provide evidence to determine whether acupotomy is an effective intervention for patients with TTS.

**Registration number:**

DOI 10.17605/OSF. IO/9PYC2 (https://osf.io/9pyc2/)

## 1 Introduction

Tarsal tunnel syndrome (TTS) is a local entrapment neuropathy that is identified as a focal compressive neuropathy of the posterior tibial nerve or 1 of its associated branches (medial plantar, lateral plantar, calcaneal nerves and Baxter's nerve^[1]^) individually or collectively under the flexor retinaculum (laciniate ligament) on the medial side of the ankle.^[2]^ It is also called posterior TTS (PTTS); PTTS is different from anterior tarsal syndrome, in which the deep peroneal nerve is compressed under the inferior extensor retinaculum (cruciate ligament) on the dorsum of the foot.^[3]^ In 1933, posttraumatic compression of the tibial nerve was described by Pollock and Davis; then, in 1960, Kopell and Thompson described the clinical manifestations of TTS.^[4]^ However, it was not until 1962 that Keck and Lam named the condition in medical literature finally and officially.^[5]^

Characteristic clinical manifestations of TTS include poorly localized paraesthesia, localized or radiating pain, burning pain, dysesthesia and hyperesthesia, and feelings of coldness that radiate from the retro-malleolar region to either the sole, heel or digits of the forefoot, or a combination of these areas.^[2,6]^ Some patients even feel as though there is a tight band around the foot. TTS usually worsens with the progression of the day and improves after relaxation, but it may manifest as cramping of the symptomatic foot. Symptoms are typically unilateral and rarely present bilaterally.^[7–9]^

TTS is relatively uncommon. The incidence of PTTS is not known; however, its incidence is less than that of carpal tunnel syndrome and that of cubital tunnel syndrome. It is easy to overlook or misdiagnosis. There is no doubt that clinical diagnosis can be identified. Causes of TTS can be classified into either intrinsic, extrinsic, or combinations of the 2.^[10]^ TTS tends to be more common in athletes such as joggers, football players, and martial arts athletes, who are subjected to prolonged weight-bearing periods inclusive of standing, walking, running or intense physical activity.^[11–13]^

It is important to take remedial measures promptly because the longer a patient has TTS, the greater the potential for lasting nerve damage. The management of TTS can involve a variety of therapeutic interventions.

Conservative management includes anti-inflammatory medication, activity modification combined with progressive mobilization exercises and naturopathy.^[14]^ Aspiration of ganglia may provide temporary benefit. Local anesthetic or corticosteroid infiltrations are recommended mainly to treat the “algetic form” of TTS to reverse any intraneural edema, but these infiltrations may increase risk.^[3]^ It is possible to decrease the pressure on the nerve to control symptoms by using orthotic shoes, activity modification, immobilization with a night splint, immobilizing braces or removable boot walker,^[14]^ but these treatments are said to worsen the symptoms in many cases. It is necessary to undergo orthopedic treatment if there is any deformity of the foot. Physiotherapy may include a variety of techniques, including bracing, stretching, icing, massage, tens and soft tissue manipulation, remedial massage therapy and ultrasound; however, evidence regarding the effectiveness of these treatments is lacking.

Surgical intervention is considered after failed nonoperative treatments, but reported success rates after tarsal tunnel decompression have varied in the literature, ranging from 44% to 96%.^[10]^ Related complications may be apparent after surgical intervention, and postoperative complications include impaired wound healing, infection, and keloid formation.^[15]^

The acupotome is a new miniature surgery instrument consisting of a bladed needle with a flat head and a cylindrical needle body that evolved from an acupuncture needle developed by Zhu Hanzhang in 1976.^[16]^ The method of utilizing acupotomes to treat abnormal, cicatricial and contractured soft tissue with microtrauma has been given the name acupotomy therapy. Acupotomy therapy is considered a minimally invasive operation in traditional Chinese medicine that combines Chinese acupuncture therapy and modern surgical principles.^[17]^ Acupotomy treats TTS by releasing the soft tissue to release pressure from the tarsal tunnel, which reduces risk, time, and cost by converting open surgery to minimally invasive surgery.

Acupotomy has been widely used clinically by practitioners of traditional Chinese medicine, orthopedics and pain departments to treat TTS in China for many years.^[18–23]^ However, from the perspective of evidence-based medicine, the safety and efficacy of acupotomy on TTS needs to be discussed. There is limited evidence in the form of systematic reviews and meta-analyses with regard to acupotomy treatment for TTS. This study will assess the effectiveness and safety of acupotomy therapy for TTS to provide evidence for further enhancing the clinical curative effects on patients with TTS.

In this study, evidence-based medicine will be used to analyze and evaluate clinical randomized controlled trials (RCTs) in patients with TTS.

## 2 Methods

### 2.1 Inclusion criteria for study selection.

#### 2.1.1 Types of studies

All RCTs examining the use of acupotomy therapy in TTS that were published in English and Chinese will be included in this systematic review and meta-analysis. Any study with a sample size less than 10 patients will be excluded from this review. Review articles, animal studies, nonclinical studies, and case reports will also be excluded. The research literature will be screened according to the criteria of the review objectives and participants, interventions, comparisons, and outcomes.

#### 2.1.2 Types of patients

Only studies in which patients have a confirmed clinical diagnosis of TTS will be included. Diagnosis requires radial ankle pain exacerbated by the Hoffmann-Tinel sign (percussing or tapping at the suspected site of compression) or the Dorsiflexion-Eversion Test (everting and dorsiflexing the ankle while maximally dorsiflexing the metatarsophalangeal joints).^[2]^ To reflect the condition's widespread nature, no restrictions will be placed upon age, sex, race, or educational status. Fracture and dislocation, muscle injury, bone tuberculosis, bone tumors, neurological symptoms and neuromuscular diseases or any systematic diseases will be excluded.

#### 2.1.3 Types of interventions.

##### 2.1.3.1 Experimental interventions

The review will involve clinical trials that focus on acupotomy treatment (there will be no limitations on the needle materials, treatment methods and treatment courses). Evaluations of acupotomy plus another treatment compared to the same treatment alone will also be included. However, studies that compare different acupotomy insertions or different forms of acupotomy will be excluded.

##### 2.1.3.2 Control interventions

Control interventions that include placebo controls, steroid injections, drug therapy, block therapy, surgery, no treatment, and acupuncture will be eligible.

#### 2.1.4 Types of outcome measures.

##### 2.1.4.1 Primary outcomes

The primary outcome measure of this systematic review will include improvement rates, functional tests, and pain relief. Evaluation will be performed by the visual analog score.

##### 2.1.4.2 Secondary outcomes

The Roles and Maudsley score will be considered as secondary outcomes.

(1) Safety: Safety will be measured by the recurrence rates of TTS, quality of life and adverse events, such as hemorrhage, serious discomfort, abscess, subcutaneous nodules, and infection.
(2) Acceptance of the measured treatment will be determined by trial exit.

### 2.2 Search methods for the identification of studies

#### 2.2.1 Electronic searches

The following electronic databases will be searched by 2 reviewers from database inception to March 2020: PubMed, Embase, Cochrane Library, Chinese literature databases, the Chinese Biomedical Literature Database, China National Knowledge Infrastructure, SinoMed, China Science and Technology Journal (VIP) and the Wanfang Database. Acupotomy RCTs examining TTS will be identified by searching these databases.

#### 2.2.2 Searching other resources

We will search the tables of contents for studies related to TTS and acupotomy, we will search the reference lists of the relevant literature, and we will search for systematic reviews to identify additional RCTs. We will also manually search relevant conference papers and will search Clinical Trials.gov and the WHO International Clinical Trials Registry Platform for new trials relevant to the topic. The search keywords or combination subject terms will include “Posterior Tarsal Tunnel Syndrome,” “Posterior Tibial Nerve Neuralgia,” “Tarsal Tunnel Syndrome,” “Tarsal Tunnel Syndromes,” “Tibial nerve entrapment,” “Tarsal Tunnel Entrapment Neuropathy,” “Tarsal Tunnel Tibial Neuropathy,” “TTS,” “Acupotomy,” “Small acupotomy,” “The small acupotomy,” “The needle knife,” “Needle-knife,” “Small needle knife,” “Acpotomoloy,” “Acupotome,” “Randomized controlled trials,” “Randomized controlled,” “Randomized,” “Controlled,” “Controlled study,” “Clinical trial,” “Controlled clinical trials,” and “Comparative study.” The accurate Chinese translation of these search terms will be used in the Chinese database. The detailed strategies for searching the PubMed database are presented in Table 1.

**Table 1:**
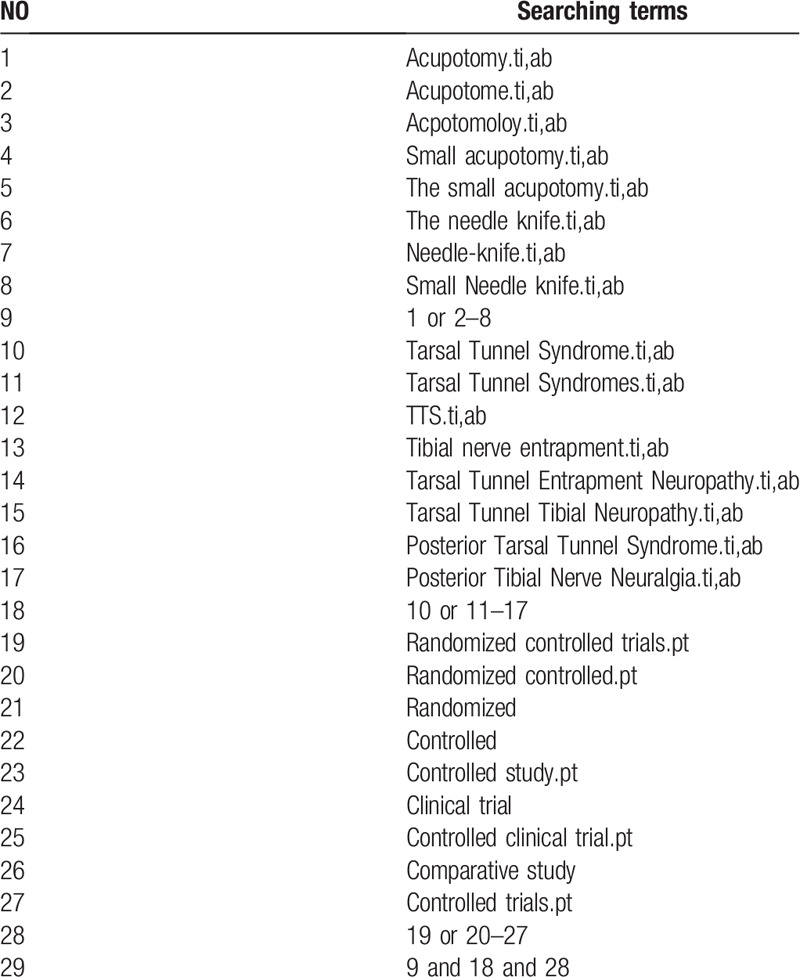
Details of the search strategy for PubMed.

### 2.3 Data collection and analysis

#### 2.3.1 Selection of studies

**Figure 1:**
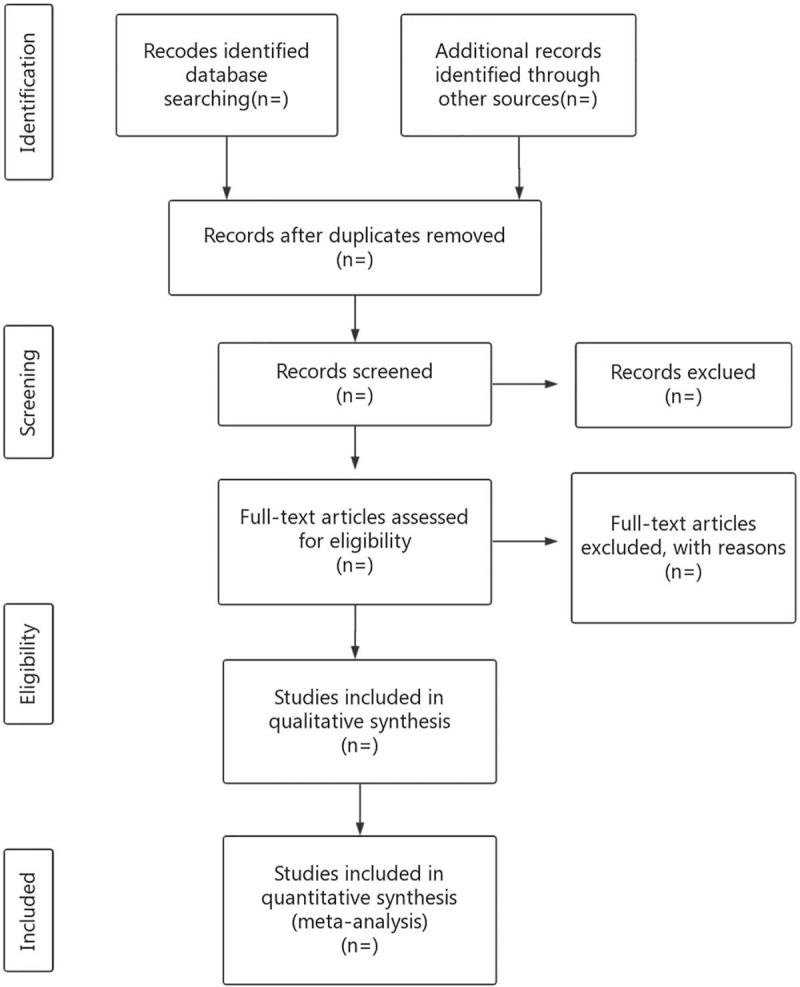
Flow diagram of the study selection process. RCT = randomized controlled trial.

The retrieved literature will be imported into the Endnote library by researchers (CS and YJ), and duplicate studies will be eliminated. Two reviewers (CS and YJ) will independently exclude articles that are noticeably below standard by reading the title and abstract. Next, the researchers will independently read the full texts, discuss the trials as a group, and contact the author to obtain details about the research to determine the eligibility of each trial (Fig. 1). The final list of articles will be converted into a Microsoft Excel format. Then, two researchers (XS and YL) will independently conduct the literature search and screening. Finally, a third independent reviewer (SL) will serve as an arbitrator and ultimately make decisions regarding inclusion.

#### 2.3.2 Data extraction and management

Data from all the selected eligible articles will be extracted by 2 independent reviewers (QZ and YL) into an Excel form. Any differences found will be resolved through discussion and recommendations from the third reviewer (SL). These data collection forms will include the reference ID, author, time of publication, randomization, participant characteristics, country, interventions, blinding, treatment indicators, follow-up, outcome indicators, research results, adverse events, and other detailed information. If necessary, we will contact the author of the trial to obtain further information.

#### 2.3.3 Assessment of the risk of bias in the included studies

Two independent reviewers (YS and ZQ) will use the Cochrane Collaboration tool to assess the risk of bias for each included trial. The following seven aspects will be assessed: random sequence generation; allocation concealment; the blinding method for patients, researchers and outcome assessors; incomplete outcome data; selective reporting; and other biases as necessary. The risk of bias will be classified as low risk, high risk and unclear.^[24]^ The third reviewer (SL) will cross-check and resolve disagreements through discussion and arbitration to obtain the results of the evaluation.

#### 2.3.4 Measures for treatment effect

We will use the relative risk or odds ratio to evaluate the enumeration data. For continuous data, the mean difference and 95% confidence interval (95% CI) will be used to assess the measurement data. If specific outcome metrics are measured using different outcome measurement scales, standardized mean difference with 95% CI will be used.

#### 2.3.5 Dealing with missing data

Researchers will contact the corresponding authors to obtain information if there are missing or incomplete data for the primary results. If the missing data are not available, we will perform the analysis based on the available data.

#### 2.3.6 Assessment of heterogeneity

Review Manager 5.3 for Windows; the Nordic Cochrane Center, Copenhagen, Denmark, will be used to evaluate the curative effect and publication bias. We will assess heterogeneously with the I2 statistic and the χ2 test in accordance with the Cochrane Handbook for Systematic Reviews of Interventions.^[25]^ We will decide whether to use a fixed effects model or a random effects model based on the heterogeneity levels of the included studies. Specifically, we will use the *χ*2 test (α = 0.1) to analyze the heterogeneity of the research results and use the *I*^2^ value to determine the significance. If *I*^2^ ≤ 50%, the statistical heterogeneity among the trials will be considered negligible, and the size of the effect will be estimated by using a fixed effects model. *I*^2^ values > 50% will be considered evidence of significant heterogeneity among the trials. After excluding the effects of significant clinical heterogeneity, we will adopt the random effects model with 95% CI for meta-analysis. If there is significant clinical heterogeneity, we will perform a subgroup or sensitivity analysis or only present descriptive statistics.

#### 2.3.7 Assessment of reporting bias

If there are more than 10 trials in the study, we will use the funnel plot to assess publication biases. We will analyze the causes for this outcome if asymmetry is observed in the funnel plot.

#### 2.3.8 Sensitivity analysis

If possible, a sensitivity analysis will be carried out to verify the robustness of the conclusions of the review. When sufficient trials are available, we will perform a sensitivity analysis to identify whether the review conclusions are robust according to the following:

(1) sample size,
(2) heterogeneity qualities, and
(3) methodological quality.

In addition, the analysis will be repeated after the exclusion of studies with low methodological quality.^[26]^

#### 2.3.9 Grading the quality of evidence

We will assess the quality of evidence with the Grading of Recommendations Assessment, Development and Evaluation framework; evidence quality will be categorized into very low, low, moderate, or high levels.^[27]^

## 3 Discussion

Acupotomy treatment is worth considering for TTS patients, as it is a minimally invasive surgery with higher acceptability and less pain. Some trials have reported that acupotomy can effectively reduce the symptoms of TTS; however, its efficacy has not been evaluated scientifically or systematically. To the best of our knowledge, there are no systematic reviews or meta-analyses of the effectiveness and safety of acupotomy on TTS that have been published. The purpose of this study was to assess the efficacy and safety of acupotomy treatment in patients with TTS. We believe our systematic review and meta-analysis will be beneficial to patients with TTS, clinicians and practitioners by providing a deeper understanding of the effectiveness of acupotomy therapy on TTS. Because there may be a risk of heterogeneity in the severity of different types of acupotomy and TTS, and the measurements and outcome assessment tools of the included studies may be different, there are some potential limitations to this review.

## Author contributions

**Investigation:** Chong Shi, Yangjing Lan.

**Methodology:** Yan Jia, Zuyun Qiu.

**Supervision:** Shiliang Li.

**Writing – original draft:** Xiaojie Sun, Qiaoyin Zhou.

**Writing – review & editing:** Yifeng Shen.
